# Mr. Hume's Test for Arsenic

**Published:** 1810-06

**Authors:** Joseph Hume

**Affiliations:** Long Acre


### *To the Editors of the Medical and Physical Journal.*

GENTLEMEN,

IN the last number of your most useful Journal there is a case detailed, and this, we are told, is not the only one, in which all the measures taken to ascertain the presence of *arsenic* failed, and where the most delicate tests were ineffectual. The importance of this subject, not merely to the chemist but to every medical practitioner, is too obvious to induce me to make any apology for the observations I may offer upon your Correspondent's case; but as there appears either some omission in the report itself or inaccuracy in the application of the means he employed, I shall very briefly give my reasons on which these doubts are founded.

Though the usual precautions to collect all the arsenic, such as the securing of both orifices of the stomach by ligatures, preserving the contents and subsequent washings, &c. still, I should think, it would have been prudent to analize, or at least to inspect, what had been rejected by vomiting; for, since this metallic substance is not very soluble, requiring more than eighty times its weight of water for solution, it is reasonable to expect a portion of the arsenic in these evacuations. As I should be sorry to provoke a controversy upon this or any other theme, I shall merely ask a few questions, which force themselves upon my notice on perusing this communication.

1. What is meant by the word *mixture*, in the experiment upon three grains of the white powder and six drachms of water?

2. In the two comparative experiments, with the above solution and with arsenic bought for the purpose, were the sky-blue and the fine green colours in the state of *precipitate* or *mixture*?

3. Was there any sub-carbonate of potash or any other *alkali* employed, previous to the admixture of sulphate of copper in solution?

4. Is not this alkali a most essential addition to any solution of white arsenic in water, in which its presence is to be proved by producing Scheele's green, or arsenite of potash?

5. Is not a *red* heat too strong to make a successful assay by means of the polished copper plates; and is it not generally recommended to envelope the arsenic with charcoal so so far as to prevent the admission of oxygen from the atmosphere during the experiment?

Though there are many very excellent processes described in elementary treatises, to prove the existence of small quantities of arsenic, yet, unless certain cautions be duly observed in their management, the operator may frequently fail in obtaining the truth and detecting the poison so certainly as to substantiate the evidence before a jury; therefore, it becomes a duty to attend most scrupulously to all the rules prescribed; to be particularly nice in the choice of the vessels, water, tests, and indeed every thing concerned with an analysis, on which may depend the character and even the life of a fellow creature.

In the Philosophical Magazine, for May, 1809, I published a new process for detecting white oxide of arsenic. This I believe was afterwards announced among the valuable lists of intelligence diffused through the medium of your Journals; but as this process cannot be too generally known, I beg here to repeat it, with some observations respecting its efficacy as a test, and the rules necessary to be observed in such an important investigation.

As an example, let us take the three grains of the suspected white powder, named by your Correspondent. Into a very clean Florence-oil flask introduce this powder; add not less than eight ounces of either *rain* or *distilled* water; heat this gradually over a lamp or a clear coal-fire till the solution begins to boil; then, while it boils, frequently shake the flask, which may be readily done by wrapping a piece of leather round its neck, or putting a glove upon the hand. To this hot solution add a single grain or two of the sub-carbonate of potash or soda, agitating the whole to make the mixture uniform. By adding the alkali *after* the solution has boiled, we are enabled to decide whether any part or the whole of the suspected powder has been dissolved; for there are instances in which an alkali increases, and others where it diminishes the solubility of various bodies.

Now, the method I would next pursue, to prove the presence of this fatal substance, is this. Into an ounce phial or a small wine glass, pour out about two table-spoonfuls of this prepared solution; then present to the mere surface of the liquid, either in the glass or phial, a piece of dry *nitrate* of *silver* or lunar caustic; if there be any white arsenic present there will instantly appear a beautiful *yellow* precipitate, which will proceed from the point of contact of of the nitrate with the fluid, and preponderate towards the bottom of the vessel as a flocculent and copious precipitate, which, I am confident, cannot be confounded with any other substance, so as to lead to a doubtful opinion.

The white oxide of arsenic is called, by most authors, *arsenious acid;* but as it shews no acid properties unless it be combined with hydrogen, that is, dissolved in water, a necessary ingredient in all acids, I am rather inclined to prefer its being classed among metallic oxides.

This excellent test, the nitrate of silver, is also extremely sensible in its operation upon *arseniate* of potash, and it serves most decidedly to distinguish this salt from the above solution or *arsenite* of potash; for the colour of this precipitate is much darker and more inclined to red or brick colour, especially after it has been suffered to collect and remain exposed to light.

I have made a number of experiments with a view to compare this test with others, for ascertaining the presence of oxide or arsenic, but particularly that with the sulphate of copper, and I have invariably found this method greatly superior, inasmuch as it produced a more copious precipitate when equal quantities were submitted to the experiment. In both cases I should recommend the dry or crystalized sulphate of copper and nitrate of silver, rather than the solution of either; and that the piece of salt should be held upon the *surface*, in order that the effect may be more perceptible.

In respect to other methods of proving the existence of arsenic, I could offer some remarks and cautions which I believe have escaped general notice; these, however, being of less consequence since a more delicate test has presented itself to me, I shall not include in this communication.

I am, &c.

JOSEPH HUME.

*Long Acre, May* 14, 1810.

