# Corrigenda

**Published:** 1810-08

**Authors:** 


Corrigenda. In the second paragraph of my last letter, after *precautions*, add, *were taken*.

